# Current Status of Artificial Intelligence-Based Computer-Assisted Diagnosis Systems for Gastric Cancer in Endoscopy

**DOI:** 10.3390/diagnostics12123153

**Published:** 2022-12-13

**Authors:** Kentaro Ochiai, Tsuyoshi Ozawa, Junichi Shibata, Soichiro Ishihara, Tomohiro Tada

**Affiliations:** 1 Department of Surgical Oncology, Faculty of Medicine, The University of Tokyo, Bunkyo-ku, Tokyo 113-0033, Japan; 2 Tomohiro Tada the Institute of Gastroenterology and Proctology, Musashi-Urawa, Saitama 336-0021, Japan; 3 AI Medical Service Inc. Toshima-ku, Tokyo 104-0061, Japan

**Keywords:** endoscopy, esophagogastroduodenoscopy, artificial intelligence (AI), gastric cancer, computer-assisted diagnosis (CAD)

## Abstract

Artificial intelligence (AI) is gradually being utilized in various fields as its performance has been improving with the development of deep learning methods, availability of big data, and the progression of computer processing units. In the field of medicine, AI is mainly implemented in image recognition, such as in radiographic and pathologic diagnoses. In the realm of gastrointestinal endoscopy, although AI-based computer-assisted detection/diagnosis (CAD) systems have been applied in some areas, such as colorectal polyp detection and diagnosis, so far, their implementation in real-world clinical settings is limited. The accurate detection or diagnosis of gastric cancer (GC) is one of the challenges in which performance varies greatly depending on the endoscopist’s skill. The diagnosis of early GC is especially challenging, partly because early GC mimics atrophic gastritis in the background mucosa. Therefore, several CAD systems for GC are being actively developed. The development of a CAD system for GC is considered challenging because it requires a large number of GC images. In particular, early stage GC images are rarely available, partly because it is difficult to diagnose gastric cancer during the early stages. Additionally, the training image data should be of a sufficiently high quality to conduct proper CAD training. Recently, several AI systems for GC that exhibit a robust performance, owing to being trained on a large number of high-quality images, have been reported. This review outlines the current status and prospects of AI use in esophagogastroduodenoscopy (EGDS), focusing on the diagnosis of GC.

## 1. Background

Artificial intelligence (AI) is a program that changes its behavior (output) in response to surrounding circumstances (input) and is constructed by simulating human intelligence. In recent years, the image recognition capabilities of AI have been improved by the emergence of machine learning methods known as deep learning, improvement in computer performance and affordability, and accumulation of large amounts of digital data. In certain areas, the performance of AI has been suggested to surpass that of humans [1,2]. In the field of medicine, AI is expected to be utilized, especially for diagnosing medical images, such as radiologic, pathologic, and endoscopic images.

Gastrointestinal endoscopy plays an essential role in the diagnosis and treatment of gastrointestinal disease. One of the challenges of endoscopy is that the examination quality varies greatly among examiners, as it requires not only expert technical skills, but also appropriate diagnostic skills for various types of gastrointestinal diseases. To solve this issue, AI-based computer-assisted detection/diagnosis (CAD) systems have been actively studied. In the field of colonoscopy, there have been many publications, including randomized control trials (RCTs) and meta-analyses, because several endoscopic AI systems for colonoscopy have been commercialized and are clinically available [3]. However, in the field of esophagogastroduodenoscopy (EGDS), the performance of AI is still insufficient for utilization in real-world clinical settings, and the CAD system for EGDS is in the developmental stage.

CAD systems can be classified into computer-aided detection (CADe), which assists endoscopists in detecting abnormal lesions during the procedure, and computer-aided diagnosis (CADx), which assists endoscopists in diagnosing the features of the lesions, such as differentiating between benign and malignant lesions, or determining the degree of progression of a lesion.

In this article, we mainly review CADe and CADx systems for gastric neoplasms, which were developed using convolutional neural networks (CNN) and are considered to be the de facto standard for deep learning-based image recognition technology.

## 2. Method

We conducted an electronic search using PubMed to identify original publications relevant to the review topic. The following search terms were used: “artificial intelligence,” “endoscopy,” “esophagogastroduodenoscopy,” “deep learning,” “neural network,” “helicobacter pylori,” “gastric cancer,” “computer-aided,” “CAD,” “CADe,” and “CADx.” Additional articles were identified through both manual search and by consulting the reference section of the included articles. Case reports, comments, and non-English publications were excluded from the review. Articles without details pertaining to methods or results were also excluded.

### 2.1. Endoscopic CAD for Gastric Cancers

Gastric cancer (GC) is the fifth most common malignant disease worldwide and the fourth most common cause of cancer mortality [4]. The 5-year survival rate of GC has been reported to be better than 95% in patients with stage I disease, whereas it dramatically decreases to 66.5% in those with stage II, and to 46.9% in cases of stage III diseases [5]. Thus, detecting GC at an earlier stage is crucial for improving the survival rate of patients. However, early stage GC resembles the background atrophic gastric mucosa, making the early diagnosis and detection of GC challenging. In fact, false-negative rates for GC in a screening endoscopy have been reported to be as high as 25.8%. Furthermore, inexperienced endoscopists tend to have higher false-negative rates [6,7,8]. To solve these issues, several CADe and CADx systems for GC are being developed (Table 1 and Table 2).

### 2.2. Endoscopic CADe for Gastric Cancers

**Figure 1:**
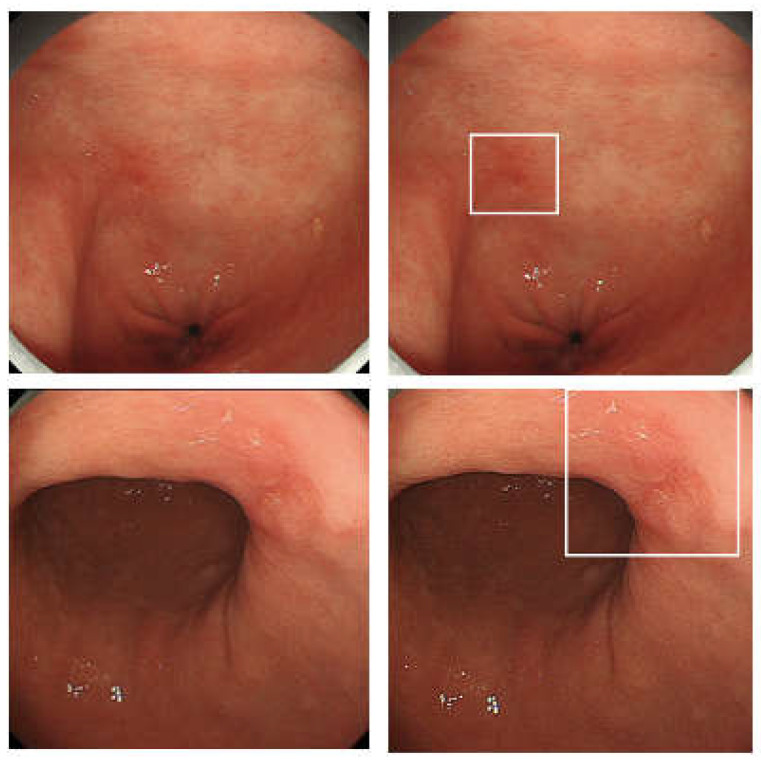
An example of CADe system for gastric cancer. The system supports the detection of gastric cancers by displaying a rectangle. CADe: computer-assisted detection.

The first AI-based endoscopic CADe system for GC was reported by Hirasawa et al. in 2018 [9] (Figure 1). The AI was trained on 13,584 endoscopic still images of GC and was validated on 2296 independent still images, and it showed a sensitivity of 92.2% for detecting GCs per image analysis. The performance of the CADe system was then validated by Ishioka et al. in 68 endoscopy videos, including those of GCs. The sensitivity of the CADe system for videos was 94.1%, which is comparable to that of still images [10].

Several retrospective studies on the performance of detecting gastric neoplasms between endoscopists and CAD systems have been reported. In the report by Ikenoyama et al., the AI showed a higher sensitivity than endoscopists (58.4% vs. 31.9%), whereas it showed a lower specificity (87.3% vs. 97.2%) and positive predictive value (PPV) (26.0% vs. 46.2%) [11]. In a cancer screening endoscopy, sensitivity is the most important parameter, and the CAD system demonstrates a preferable performance. However, the specificities of these CAD systems were comparatively low, and the specificity reported by Hirasawa et al. in a similar study was also as low as 30.6%, suggesting that a low specificity is one of the issues associated with the CADe systems for GC. One of the reasons the CADe system reported by Hirasawa et al. had a low specificity was the high rate of the false-positive detection of gastric ulcers (GU) as GC [11]. Namikawa et al. trained the CAD system using 13,584 GC images and 4453 GU images to improve the false-positive rate owing to the misidentification of GU as GC [12]. The performance of the CAD system was validated on 739 GC and 720 GU images, and the specificity successfully improved to 99.0%, whereas the sensitivity remained at 93.3%.

A few prospective studies comparing the performance of CADe for GCs between endoscopists and CAD systems have also been reported. In 2019, Luo et al. first reported a multicenter case-control study evaluating the performance of the CADe system for upper gastrointestinal tract cancers, named GRAIDS. They reported an accuracy of 92.7% in detecting both gastric and esophageal cancers. They also compared the diagnostic performance of the CADe system and those of endoscopists and reported that the performance of the CADe system was comparable to that of endoscopists with >10 years of experience (94.2% vs. 94.5% in sensitivity) and superior to those of competent (85.8%) and trainee (72.2%) endoscopists [13]. Niikura et al. compared the GC detection performance of expert endoscopists and CAD using retrospective data consisting of 500 patients (including 100 GC cases). In this study, background information (e.g., prevalence of early/advanced GC, patients’ H. Pylori infection status, etc.) was matched between the two conditions using a computer-based system. The reported sensitivity for the CAD system was 100% (49/49 cases), comparable to the expert endoscopists’ sensitivity of 94.1% (48/51 cases) [14]. These reports suggest that AI may have the potential to fill the gap in diagnostic performance between expert and non-expert endoscopists.

Wu et al. reported a series of studies using their CAD system for gastric neoplasms named ENDOANGEL, which was created based on deep reinforcement learning (DRL) and CNN, and has multiple functions, including the detection of GC and pre-cancerous lesions, differentiation of cancerous and non-cancerous lesions, prediction of tumor invasion depth, and anatomical detection of the upper gastrointestinal tract. In a tandem RCT that compared the miss rate of gastric neoplasms between routine EGDS (routine-first, n = 905) and EGDS with the CAD-first group (AI-first, n = 907), they reported that the miss rate of neoplasms in the AI-first group was significantly lower than that in the routine-first group (6.1% vs. 27.3%, *p* = 0.015), suggesting that the use of AI could reduce the number of overlooked gastric neoplasms [15]. According to another prospective study that compared the detection of early gastric cancer (EGC) using white light imaging (WLI) between the CADe system and endoscopists, the AI outperformed the endoscopists in specificity (93.2% vs. 72.3%, *p* < 0.01), accuracy (91.0% vs. 76.9, *p* < 0.01), and PPV (90.0% vs. 76.9%, *p* = 0.013) [16]. They conducted another trial in a setting that considered the clinical use of AI. In this prospective trial of a total of 2010 patients, endoscopists performed EGDS using ENDOANGEL, and the performances of ENDOANGEL as CADe and CADx systems for gastric neoplasms were evaluated. They reported a sensitivity of 91.8% and specificity of 92.4% for the detection of gastric neoplasms, indicating that the AI may be useful in real-world clinical settings [17].

These reports suggest that CADe for GC may have a comparable performance to that of expert endoscopists and may even surpass that of inexperienced endoscopists in detecting GCs. EGDS is performed by various endoscopists at different levels; therefore, it is expected that AI will equalize their performance to that of specialists regardless of the skill of the endoscopist.

### 2.3. Endoscopic CADx for Gastric Cancers

Once a gastric lesion is detected, differentiation into neoplastic and non-neoplastic is necessary. Currently, image-enhanced technologies, such as narrow band imaging (NBI) and blue laser imaging (BLI) or magnified endoscopy, have been developed to help differentiate gastric lesions. NBI and BLI were equipped with commercial endoscopy systems in 2006 and 2012, respectively. The combination of a magnifying endoscopy and these image-enhancing technologies has been widely used in clinical practice to visualize microvascular and mucosal surface microstructures with a higher contrast. The usefulness of the vessel plus surface classification system (VSCS) or a diagnostic algorithm for early gastric cancer using magnified NBI (M-NBI) to differentiate gastric neoplasms has been reported [18,19,20,21]. CADx systems for differentiating cancerous/non-cancerous lesions have also been developed, and most are based on enhanced images.

Li et al. and Ueyama et al. reported a high diagnostic accuracy of their CADx systems for GC based on M-NBI images, with sensitivities of 95.4% and 98%, and specificities of 71.0% and 100%, respectively [22,23]. Horiuchi et al. reported a sensitivity of 91.18% and a specificity of 90.64% of their CADx systems for GC on M-NBI still images [24]. They also validated their CADx system using 174 videos of M-NBI and reported a sensitivity of 87.4% and specificity of 82.8%, indicating that it may be feasible for real-time differentiation during EGDS [25]. Furthermore, they compared the performance of the CADx system with that of endoscopists and reported that the performance of the CADx system was comparable to that of expert endoscopists.

Hu et al. investigated whether endoscopists’ diagnostic performance could be improved by the CADx system. While the diagnostic accuracy of the CADx system is similar to that of expert endoscopists, expert endoscopists that used the CADx system demonstrated improved performance in sensitivity (76.7% vs. 87.4%) and negative predictive value (74.5% vs. 83.6%) [26]. This report suggests that the CADx system for GC could benefit not only non-experts but also the experts.

Wu et al. prospectively evaluated the performance of ENDOANGEL, which had been developed using M-NBI images, and reported a sensitivity of 100% and a specificity of 82.54%, which were equivalent to those of endoscopists [15].

While all of the aforementioned reports were based on enhanced images, Wu et al. reported a CADx system based on WLI [16]. They prospectively attempted to differentiate neoplastic/non-neoplastic lesions using their CADx system and reported a sensitivity of 92.9% and a specificity of 91.7%, suggesting that AI can be used to differentiate with a high accuracy, even with WLI. Recently, Ishioka et al. developed a CADx system for EGCs, called Tango. They compared the performance of the CADx system with that of endoscopists, using a dataset comprising only EGCs and benign lesions. Tango demonstrated a superior sensitivity over even specialists (84.7% vs. 65.8%) [27].

AI has also been reported to differentiate between lesions other than GC. Yuan et al. reported an AI capable of multiclassification not only for GC. The AI was trained on 29,809 images containing various lesions and tested on 1579 images to classify lesions into EGC, advanced GC, submucosal tumor, polyp, ulcer, erosion, and normal mucosa [28]. The overall accuracy was 85.7%, which was equivalent to that of senior endoscopists (85.1%) and higher than that of junior endoscopists (78.8%).

### 2.4. Endoscopic CADx for Diagnosing Various Features of Gastric Cancers

Endoscopic submucosal dissection (ESD) is the preferred treatment option for EGCs because, compared to surgery, it is less invasive, achieves superior postoperative quality of life, and is more cost-effective. However, its indication is limited to lesions with a low risk of lymph node metastasis (LNM). In the Japanese guidelines, the indication of endoscopic resection for GCs is based on multiple factors, including tumor diameter, depth, differentiation status, and the presence of ulcers [11].

Regarding tumor depth, GCs with invasion depths of M and SM1 (<500 μm) are considered to have a low risk of LNM, while those with depths of SM2 and deeper have a high risk of LNM, and surgical intervention is recommended for such tumors in the Japanese GC treatment guidelines [11]. Endoscopic resection is also recommended for early stage localized disease (cTis-cT1a) in the National Comprehensive Cancer Network guidelines [29].

In current clinical practice, the tumor invasion depth is predicted based on macroscopic features using conventional endoscopy or endoscopic ultrasonography (EUS). With conventional endoscopy, various morphological features have been reported as predictors of the tumor invasion depth [30]. Reported indicators of EGC deeper than SM1 observed with WLI include pathomorphological changes at the tips of converging folds, a tumor diameter greater than 30 mm, marked erythema, surface irregularities, marginal elevation with and without submucosal tumor-like features, and trapezoid elevation [31,32,33,34,35]. However, owing to its subjective nature, an accurate prediction remains challenging.

Yoon et al. developed an AI system for assessing the tumor invasion depth (T1a or T1b) with non-magnified WLI and reported a sensitivity of 79.2% and a specificity of 77.8% [36]. Similarly, Cho et al. developed a CADx system to diagnose the tumor depth (Tis/T1 or T2) in non-magnified WLI and reported a sensitivity of 80.4% and a specificity of 80.7% [37].

While these CADx systems were for non-magnified WLI, Nagao et al. evaluated the performance of CADx to diagnose the tumor depth using enhanced images, including NBI and indigo-staining chromoendoscopy [38]. They reported a sensitivity, specificity, and accuracy of 75.0%, 100.0%, and 94.3%, respectively, with NBI; and 87.5%, 100.0%, and 95.5%, respectively, with indigo-stained images, which were comparable to those with WLI.

Zhu et al. and Tang et al. compared the performance of AI and endoscopists in predicting the tumor invasion depth. Zhu et al. used 790 images to develop a CADx system to differentiate the invasion depth of GCs (M or SM1/deeper than SM1) and compared its performance with that of endoscopists using 203 independent validation images. They reported that the performance of the CADx system outperformed those of both junior and experienced endoscopists in accuracy (AI, 89.16%; junior endoscopists, 66.17%; and experienced endoscopists, 77.46%) and specificity (AI, 95.56%; junior endoscopists, 56.71%; and experienced endoscopists 70.74%) [39]. Tang et al. reported that the CADx system showed an accuracy of 88.2%, a sensitivity of 90.5%, and a specificity of 85.3% for differentiating mucosal/submucosal invasion in WLI images. They also reported that the diagnostic performance of endoscopists improved by using a CADx system not only in novice endoscopists (accuracy 74.0% vs. 84.6%, *p* < 0.001; sensitivity 81.1% vs. 85.7%, *p* = 0.018; specificity 65.2% vs. 83.3%, *p* < 0.001) but also in expert endoscopists (accuracy 79.8% vs. 85.5%, *p* < 0.001; sensitivity 84.3% vs. 87.4%, *p* = 0.018; specificity 74.2% vs. 83.0%, *p* < 0.001) [40].

The AI reported by Nam et al. was a multistep model that first detected gastric lesions from endoscopic images, then classified them as GU, early GC, or advanced GC, and finally predicted the tumor invasion depth (T1a/T1b) for lesions classified as early GC [41]. The performance of the AI was compared with the EUS for the invasion depth prediction in this study, which reported that the AI had a better area under the receiver operating characteristic curve (AUC) than the EUS performed by experts (0.73 vs 0.56) in the test with the external validation dataset.

Tumor differentiation status is also an important factor in determining indications for endoscopic resection. According to Japanese guidelines, endoscopic resection for undifferentiated-type GC is prescribed as an “expanded indication” because of the lack of sufficient evidence of long-term outcomes [42]. Regarding the differentiation status, undifferentiated types are associated with a high risk of LNM. Undifferentiated EGCs tend to have flat or depressed macroscopic features and are less likely to show raised features, whereas differentiated EGCs have both depressed and raised types. In addition to these macroscopic findings, microstructures with M-NBI findings have been used to predict the differentiation status [19,43].

Ling et al. reported a CADx system that predicts the differentiation status of lesions using M-NBI images. The CADx system showed a better accuracy than those of endoscopists (86.2% vs. 69.7%) [44].

A prospective study by Wu et al. using ENDOANGEL demonstrated a tumor invasion depth diagnosis and differentiation status prediction. This prospective study was conducted in a practical setting in which the AI detected gastric lesions using non-magnified WLI, then differentiated them into cancerous/non-cancerous lesions using M-NBI images, and, finally, the invasion depth (M or SM) and differentiation status (differentiated or undifferentiated). They reported that the performance of ENDOANGEL was comparable to those of expert endoscopists in predicting the invasion depth (sensitivity 70.0% vs. 56.7%, specificity 83.33% vs. 76.2%, accuracy 78.57% vs. 83.02%) and differentiation status (sensitivity 50.0% vs. 46.83%, specificity 80.00% vs. 71.89%, accuracy 71.43% vs. 71.89%) [16].

### 2.5. Endoscopic CADx for Helicobacter Pylori Infection

The first CNN-based computer-assisted diagnosis (CADx) system for gastric lesions in EGDS images was a CADx system for determining the presence of HP infection.

HP infection is one of the most important risk factors for gastric cancer (GC) [3]. HP infection causes atrophic gastritis, which progresses as the exposure period increases. It has also been shown that patients with severe atrophic gastritis have a higher risk of GC compared with those who have mild gastritis. Meanwhile, the eradication of HP has been suggested to decrease the incidence of GCs, as it halts the progression of gastritis. Thus, during the routine or screening EGDS, it is important to diagnose atrophic gastritis at an early stage [45]. However, to diagnose the presence or absence of HP-related atrophic gastritis requires a lot of training for endoscopists. Several AI-based CADx systems for determining HP-related atrophic gastritis have been developed (Table 3). Shichijo et al. first reported a CADx system for the presence of HP infection in 2017, which was trained on 32,208 endoscopic still images with or without HP infection, and the performance of the AI was validated on 11,481 independent still images. They reported the accuracy, sensitivity, and specificity of diagnosing the presence of current or past HP infection as 87.7%, 88.9%, and 87.4%, respectively [46], and it outperformed novice endoscopists. Although HP eradication cases are less likely to have GCs, it is much more challenging to endoscopically differentiate HP-eradicated cases from HP cases, even for skilled endoscopists [47]. The authors updated the CADx system using 98,564 endoscopic images, including 845 HP-eradication cases. It was validated on 847 independent cases (23,699 images), and the accuracies were 80% for HP-negative images, 48% for HP-positive images, and 84% for HP-eradicated images, respectively, in per-images analysis [48].

Nakashima et al. developed an AI diagnosis for HP infection using white light imaging (WLI) and linked color imaging (LCI) [49]. In per-patient analyses, the accuracies to diagnose uninfected images were 75.0% with WLI and 84.2% with LCI. The accuracy in diagnosing infected images was 77.5% with WLI and 82.5% with LCI. While the accuracy in diagnosing posteradication images was 74.2% with WLI and 79.2% with LCI.

While all these reports were retrospective and validated with still images, Nakashima et al. and Xu et al. evaluated the performance of AI using video images. Nakashima et al. trained their AI using WLI and LCI still images from 395 patients and validated it using endoscopic videos of the gastric lesser curvature. They reported an accuracy of 84.2% for uninfected cases, 82.5% for currently infected cases, and 79.2% for posteradication cases [50]. Xu et al. prospectively tested the performance of ENDOANGEL in diagnosing gastric atrophy, and its accuracy was 87.8% in video images [51]. These results suggest that the clinical application of AI for detecting HP infection may be feasible.

### 2.6. Endoscopic CAD for Quality Assurance

The AI systems described above were designed to detect and differentiate lesions in endoscopic images. However, its performance cannot be fully demonstrated unless all parts of the stomach are observed appropriately. In this regard, AI-based endoscopy support approaches differ from just detecting/diagnosing abnormal lesions, but developments have been made to reduce blind spots during the examination and to monitor the quality of endoscopy (Table 4).Wu et al. developed an AI system named WISENSE, the predecessor of ENDOANGEL, which can detect the anatomical part of the observation by learning 24,549 normal images of different parts of the stomach with the aim of reducing blind spots during examination [52]. They evaluated the performance of this AI through RCT and reported that the WISENSE-user group showed significantly lower blind spot rates than the non-user group (5.86% vs. 22.46%, *p* < 0.001) [53]. In a multicenter RCT conducted to evaluate the blind spot rates between those with or without ENDOANGEL, the ENDOANGEL-assisted group had significantly fewer blind spot rates (5.35% vs. 9.82%, *p* < 0.001) and a longer inspection time than the non-assisted group, although a longer inspection time was observed in the former group (5.40 min vs. 4.38 min, *p* < 0.001) [54]. Furthermore, ENDOANGEL correctly predicted all five GCs with a per-lesion accuracy of 84.7%, sensitivity of 100%, and specificity of 84.3%.

Similarly, the endoscopic AI system named IDEA, which was developed by Li et al., is capable of monitoring blind spots and provides an operation score during EGDS. The operation score is then graded according to the observed part of the stomach with a higher score, implying a lower blind spot rate. The results of a multicenter prospective study using IDEA showed that the operation score output of IDEA significantly correlated with higher GC detection rates, indicating that AI assistance may improve the quality of examination [55]. These results suggest that AI may also be useful for the quality control of upper endoscopy.

## 3. Discussion

In recent years, CNN-based AI systems have been mainly used for image recognition, and their capabilities have partly surpassed those of humans. In the medical field, AI systems have been applied to radiologic image recognition, such as X-rays, computed tomography (CT), and magnetic resonance imaging (MRI). Recently, it has been applied to endoscopic image recognition. However, endoscopic image recognition is considered to be more challenging than radiographic image recognition because endoscopic images are affected by multiple conditions, including the distance, angle, and clearance of the region of interest. Therefore, the development of CAD systems for endoscopy may be more difficult than the development of CAD systems for radiologic images. Recent progress in computer technology, in which copious digitized, high-resolution images are available as big data, has helped improve AI performance. State-of-the-art CNN technologies that can analyze endoscopic imagery with a high accuracy have also been reported on [56]. These technological advances resulted in the development of high-performance CAD systems for endoscopy. This paper presents a literature review of recent advances in CAD systems for EGDS. This review presents the most up-to-date discussion of endoscopic AI for GC diagnosis in upper GI. It is also the first review to report on the specific AI modalities of CADe, CADx, and CAD for quality assurance. As shown in this review, in the past few years, rapidly increasing reports of AI for GC with a robust performance achieved by learning a large number of high-quality images have been published. For CADe systems, a number of systems with sensitivities above those of novice endoscopists and equivalent to those of experts have been reported. RCTs have also shown that the use of the CADe system lowers the miss rate of GC. Regarding CADx, although RCTs have not yet been reported, many have shown expert-level performance. These results suggest that a CADx system could improve the diagnostic performance of non-expert endoscopists and bring them to the expert level.

However, there are fairly limited reports of CAD systems that perform better than the experts; thus, it remains to be seen whether AI can also be useful to the experts. There have also been a few reports of CAD systems that focus exclusively on EGC, which is far more difficult to detect/diagnose than advanced GCs. Furthermore, it should be noted that the previously reported specificity of CAD systems tends to be lower than that of endoscopists. In promoting the social implementation of AI, low specificity may be a drawback that leads to an increase in over diagnosis, leading to an increase in unnecessary additional tests, such as biopsies, which may increase medical costs and morbidity. The reimbursement and cost-effectiveness of AI are important concerns. In the field of colonoscopy, there are a few reports that suggest that the use of the CADe system may be cost-effective in reducing the incidence of colorectal cancer through improvement in adenoma detection and by reducing unnecessary polypectomy through accurate polyp differentiation [57,58]. Regarding the use of CAD systems for EGDS, its cost-effectiveness has not been fully verified so far; therefore, further research is expected in the near future. In addition, most studies on endoscopic AI for GC are from Asian countries, such as Japan and China, which may be due to the high prevalence of HP in these areas [59]. Thus, it is also necessary to verify whether these endoscopic AIs can be used with similar outcomes in Western populations.

AI for GC is being developed not only for cancer detection and differentiation but also for multifaceted approaches, such as tumor invasion depth prediction, prediction of HP infection, and monitoring examination quality. It is expected that these technologies will greatly contribute to the early detection and diagnosis of GCs for appropriate treatment selection.

## 4. Conclusions

The latest research and development trends of CNN-based endoscopic AI for GCs have been outlined. Most reports on AIs have shown that AIs have a better diagnostic performance than non-expert endoscopists and their performance is equivalent to those of the experts. Most AI systems presented in this review are based on training data annotated by expert endoscopists, indicating that endoscopic AI is the culmination of endoscopists’ wisdom. Future studies are required to evaluate the usefulness of endoscopic AI in clinical settings.

**Table 1:** Summary of CADe for gastric cancer.

Study Design	Reference, Year	Modality	Training Dataset	Validation/Test Dataset	AUC	Accuracy (%)	Sensitivity (%)	Specificity (%)
Retrospective	Hirasawa, 2018 [9]	WLI, CE, NBI	51,558 images (13,584 GC images)	296 GC images	n/a	n/a	92.2	n/a
	Yoon, 2019 [36]	WLI	11,539 images (1705 GC images)	11,539 images (1705 GC images)	0.981	n/a	91	97.6
	Ishioka, 2019 [10]	WLI, CE, NBI	51,558 images (13,584 GC images)	68 videos with GC	n/a	n/a	94.1	n/a
	Ikenoyama, 2021 [11]	WLI, CE, NBI	51,558 images (13,584 GC images)	2940 GC images of 140 patients	0.757	n/a	58.4	87.3
	Nam, 2022 [41]	WLI	1009 images (110 GU, 620 EGC, 279 AGC)	112 images (internal test), 245 images (external test)	0.78 (internal test), 0.73 (external test)	n/a	n/a	n/a
	Niikura, 2022 [14]	WLI	51,558 images (13,584 GC images)	500 patients (51 AGC, 49 EGC patients)	n/a	n/a	100	n/a
Prospective	Luo, 2019 [13]	WLI	141,570 images (26,172 GC/EC images)	66,750 images (4317 GC/EC images)	0.974	92.7	94.6	92.6
	ENDOANGEL							
Prospective	Wu, 2022 [16]	WLI	24,704 images (15,341 GC); ENDOANGEL-CNN1a (detection module)	100 lesions from 96 patients	n/a	91	87.81	93.22
	Wu, 2022 [17]	WLI	21,000 images (15,341 GC); ENDOANGEL-LD CNN1	internal test1: 1198 images (1000 GC), internal test2: 5488 images (338 neoplastic), external test: 15,886 images (774 neoplastic)			98.3 (internal test1), 96.9 (internal test2), 95.6 (external test), 100 (videos)	98.4 (internal test1), 90.6 (internal test2) and 90.8 (external test)
RCT	Wu, 2021 [15]	WLI	18,579 images (12,447 GC)	1012 patients (93 patients with GC)	Findings: The gastric neoplasm miss rate was significantly lower in the AI-first group than in the routine- first group (6.1% vs 27.3%, *p* = 0.015).			

CADe: computer-assisted detection; GC: Gastric cancer; RCT: Randomized control trial; AUC: Area under the curve; WLI: White light imaging; CE: Chromoendoscopy; NBI: Narrow band imaging; BLI: Blue laser imaging; GU: Gastric ulcer; EGC: Early gastric cancer; AGC: Advanced gastric cancer.

**Table 2:** Summary of CADx in gastric cancer.

	Study Design	Reference, Year	Modality	Training Dataset	Validation/Test Dataset	AUC	Accuracy (%)	Sensitivity (%)	Specificity (%)
CADx-neoplastic/non-neoplastic	Retrospective	Li, 2020 [22]	M-NBI	2088 images (1702 EGC)	341 images (170 EGC)	n/a	90.91	91.18	90.64
		Horiuchi, 2020 [24]	M-NBI	2570 images (1492 EGC)	258 images (151 EGC)	0.85	85.3	95.4	71
		Horiuchi, 2020 [25]	M-NBI	2570 images (1492 EGC)	174 videos (87 with EGC)	0.8684	85.1	87.4	82.8
		Namikawa, 2020 [12]	WLI, NBI	18,410 images (2649 GC, 4826 GU)	739 EGC and 720 GU images	n/a	99.0 (GC), 93.3 (GU)	99.0 (GC), 93.3 (GU)	93.3 (GC), 99.0 (GU)
		Ueyama, 2021 [23]	M-NBI	5574 images (3797 EGC)	2300 images (1430 EGC)	n/a	98.7	98	100
		Hu, 2021 [26]	M-NBI	Images from 170 patients with EGC	73 patients (Internal test), 52 patients (External test)	0.808 (Internal test), 0.813 (External test)	77.0 (Internal test), 76.3 (External test)	79.2 (Internal test), 78.2 (External test)	74.5 (Internal test), 74.1 (External test)
		Nam, 2022 [41]	WLI	1009 images (110 GU, 620 EGC, 279 AGC)	112 images (internal test), 245 images (external test)	Internal test 0.89 External test 0.82	Internal test GU 95, EGC 89, AGC 93 External test: GU 86, EGC 79 AGC 79	Internal test GU 63 EGC 94 AGC 90 External test GU 68 EGC 77 AGC 56	Internal test GU 98 EGC 82 AGC 94 External test GU 50 EGC 89 AGC 47
		Yuan, 2022 [28]	WLI	29,809 images	1579 images	n/a	85.7	n/a	n/a
		Ishioka, 2022 [27]	WLI	40,162 images (18,027 EGC)	315 mages (150 EGC)	n/a	70.8	84.7	58.2
		ENDOANGEL							
	Prospective	Wu, 2022 [16]	M-NBI	8301 WLI images (4442 neoplastic images); ENDOANGEL-CNN1b (WLI), 4667 M-NBI images (1950 EGC images); ENDOANGEL-CNN2 (M-NBI)	100 lesions from 96 patients	n/a	89	100	82.54
		Wu, 2022 [17]		9824 images (5359 neoplastic images); ENDOANGEL-LD CNN2	Internal test1: 1198 images (1000 abnormal), Internal test2: 5488 images (338 neoplastic) External test: 15,886 images (774 neoplastic) 100 videos (38 neoplastic)	0.960 (internal test1),	Internal test1: 86.0 Internal test2: 88.8 External test: 88.6 Videos: 72.0	Internal test1: 94.0 Internal test2: 92.9 External test: 91.7 Videos: 100	Internal test1: 84.0 Internal test2: 88.8 External test: 88.2 Videos: 53.2
CADx-Invasion depth, pathological status	Retrospective	Kubota, 2012 [60]	WLI	902 GC images	902 GC images	n/a	64.7	n/a	n/a
		Yoon, 2019 [37]	WLI	1750 GC images	1705 GC images	0.851	n/a	79.2	77.8
		Zhu, 2019 [39]	WLI	790 GC images	203 GC images	0.94	89.16	76.47	95.56
		Nagao, 2020 [38]	WLI, CE, NBI	13,628 GC images	2929 GC images	0.959 (WLI), 0.9048 (NBI), 0.9491 (CE)	94.49 (WLI), 94.30 (NBI), 95.50 (CE)	84.42 (WLI), 75.00 (NBI), 87.50 (CE)	99.37 (WLI), 100 (NBI), 100 (CE)
		Cho, 2020 [37]	WLI	2899 images	206 images	0.887	77.3	80.4	80.7
		Tang, 2021 [40]	WLI	3407 images from 666 GC patients	228 images	0.942	88.2	90.5	85.3
		Ling 2021 [44]	M-NBI	2217 GC images	1870 GC images	n/a	86.2	Differentiated: 88.6, Undifferentiated: 78.6	Differentiated: 78.6, Undifferentiated: 88.6
		Nam, 2022 [41]	WLI	1009 images (110 GU, 620 EGC, 279 AGC)	112 images (internal test), 245 images (external test)	Internal test: 0.78, External test: 0.73	Internal test: 77, External test: 72	Internal test: 86, External test: 73	Internal test: 66 External test: 94
	Prospective	Wu, 2022 [16]	WLI, M-NBI	3407 WLI images; ENDOANGEL-CNN3 (invasion depth), 2217 M-NBI images (1131 differentiated a 1086 undifferentiated); ENDOANGEL-CNN4 (differentiation status)	28 lesions from 28 patients	n/a	78.6 (submucosal invasion), 71.4(undifferentiated EGC)	70.0 (submucosal invasion), 50.0 (undifferentiated EGC)	83.3 (submucosal invasion), 80.0 (undifferentiated EGC)

CADe: Computer-assisted detection; CADx: Computer-assisted diagnosis; GC: Gastric cancer; AUC: Area under the curve; WLI: White light imaging; CE: Chromoendoscopy; NBI: Narrow band imaging; M-NBI: Magnified narrow band imaging; BLI: Blue laser imaging; GU: Gastric ulcer; EGC: Early gastric cancer; AGC: Advanced gastric cancer.

**Table 3:** Summary of CADx for *Helicobacter pylori* infection.

Study Design	Reference, Year	Modality	Training Dataset	Validation/Test Dataset	AUC	Accuracy (%)	Sensitivity (%)	Specificity (%)
Retrospective	Shichijo, 2017 [47]	WLI, CE, NBI	32,208 images from 1750 patients (753 HP-positive)	11,481 images from 397 patients (72 HP-positive)	0.93	87.70%	88.9	87.4
	Shichijo, 2019 [48]	WLI	98,564 images from 5236 patients (742 HP positive, 3649 HP negative, and 845 HP eradicated)	23,699 images from 847 patients (70 positive, 493 negative, and 284 eradicated)	n/a	80 (HP negative), 84 (HP eradicated), 48 (HP positive)	n/a	n/a
	Zheng, 2019 [61]	WLI	11,729 images from 1959 patients (847 HP positive)	3755 images form 452 patients (310 HP positive)	0.97	93.8	91.6	98.6
	Guimarães, 2020 [62]	WLI	200 images (100 HP positive)	70 images (30 HP positive)	0.981	92.9	100	87.5
	Zhang, 2020 [63]	WLI	A total of 5470 images (3042 with atrophic gastritis), 70% for training and 30% for testing		0.99	94.2	94.5	94
Prospective	Itoh, 2018 [64]	WLI	149 images (70 HP positive)	30 images (15 HP positive)	0.956	n/a	86.7	86.7
	Nakashima, 2018 [49]	WLI, BLI, LCI	2592 images from 162 patients (75 HP positive)	60 patients (30 HP-positive)	0.66 (WLI), 0.96 (BLI), 0.95 (LCI)	n/a	66.7 (WLI), 96.7 (BLI), 96.7 (LCI)	60.0 (WLI), 86.7 (BLI), 83.3 (LCI)
	Nakashima, 2020 [50]	WLI, LCI	12,887 images from 395 patients (138 HP positive, 141 HP negative, 116 HP eradicated)	120 videos (40 HP positive, 40 HP negative, 40 HP eradicated)	0.82 (LCI, HP positive), 0.90 (LCI, HP negative), 0.77 (LCI, HP eradicated)	HP Positive 77.5 (WLI), 82.5 (LCI) HP negative 75.0 (WLI), 84.2 (LCI) HP eradicated 74.2 (WLI), 79.2 (LCI)	HP Positive 60.0 (WLI), 62.5 (LCI) HP negative 95.0 (WLI), 92.5 (LCI) HP eradicated 35.0 (WLI), 65.0 (LCI)	HP Positive 86.2 (WLI), 92.5 (LCI) HP negative 65.0 (WLI), 80.0 (LCI) HP eradicated 93.8 (WLI), 86.2 (LCI)
	Xu, 2021 [51]	M-NBI, M-BLI	354 patients	77 videos	0.878	87.8	96.7	73

CADx: computer-assisted diagnosis; HP: Helicobacter pylori; AUC: Area under the curve; WLI: White light imaging; CE: Chromoendoscopy; NBI: Narrow band imaging; BLI: Blue laser imaging.

**Table 4:** Summary of CAD for examination quality assurance.

Reference, Year	Study Design	Application	Modality	Training Dataset	Validation/Test Dataset	Findings
Wu, 2019 [52]	Retrospective	Classification of observed location	WLI	24,549 images	170 images	Accuracy: 90 (into 10 parts), 65.9 (into 26 parts)
Wu, 2019 [53]	RCT	Monitoring blind spots	WLI	34,513 images	107 videos	Accuracy: 90.0% Sensitivity: 87.5%, Specificity 95.0%
Li, 2022 [55]	Prospective	Monitoring EGD quality	WLI	170,297 images and 149 videos	17,787 patients	AI out put the EGD quality monitoring scores. The cancer detection rate (r = 0.775) and early cancer detection rate (r = 0.756) were positively correlated with total score.

CAD: computer-assisted detection/diagnosis; GC: Gastric cancer; RCT: Randomized control trial; AUC: Area under the curve; WLI: White light imaging; EGC: Early gastric cancer.

## Data Availability

Data are contained within the article.
